# Modern Sociology

**Published:** 1899-08-19

**Authors:** 


### Modern Sociology.

### WHAT WOMEN CAN DO.

The establishment of the Central Bureau for the Employment of Women, in connection with the National Union of Women Workers, is a step, and a most important one, in the direction of showing women in what ways they can best earn their living. The committee of the bureau shows a long list of names of those who are known to have helped to raise the industrial status of women. The Countess of Dudley is chairman, and with her are associated Lady Ulrica Duncombe, Mrs. Sophie Bryant, Mrs. Creighton, Mrs. George Cadbury, Sir Walter Besant, and others almost as well known; while the fact that Miss Margaret Bateson is the honorary secretary of itself proves that those who have undertaken the conduct of the bureau know women's needs and women's capacities. The bureau is largely intended for those who have not been brought up from their youth to any special occupation, but who, when they have arrived at womanhood, find themselves, either from love of work or need of money, desirous of entering the industrial arena. Much of the work done is of an advisory nature. Applicants write, not to ask for aid in finding a particular kind of situation, but to find out what kind of situation they are fit for. For such advice a fee of only sixpence is charged, though the reply may entail considerable trouble to the officers of the bureau. In this, which is one of its main functions, the bureau can never hope to become self-supporting, and for this reason those who have founded it ask for subscriptions from the public. A small donation to its funds might often do more to relieve us of that most pitiful incubus of civilisation, the "indigent gentlewoman," than a much larger sum devoted to particular cases. The hon. treasurer is Sir Robert Giffen, K.C.B., 9, Bina Gardens, South Kensington, S.W.

Besides giving advice, the bureau fulfils the ordinary functions of a register. Applicants for situations pay a fee of 1s. on entering their names, and sums varying from 2s. to 7s. 6d. on obtaining a situation through the agency of the bureau, according to the salary they are to receive. As it has been in existence only since January, 1898, the work of the bureau can hardly be fully estimated yet, but already it has found good situations for a number of women who, but for having it as intermediary, might have been forced to be content with much less remunerative ones. Among these are mentioned manageress in a manufactory, at a salary of £120 per annum; housekeeper in a college for women, cashier to a women's club, housekeeper to a dressmaker, teacher of evening classes, and private resident gardener. In short, anyone with a specialty in labour to sell can in all probability get a better price for it through the agency of the Central Bureau.

Of course the trouble is with those women who have no specialty, who have been trained for nothing. But if they have an inclination for any kind of work they will get information as to where they can best train for it, and also as to the chances of making a living by it when trained. Some of the most likely professions are mentioned in the report of the bureau, but we think the list could be added to considerably. Particular attention has been given to the requirements for secretaries and clerks. There is a steady demand for trained women for such posts, the necessary qualifications being good shorthand, type writing (with practical experience of two or three machines), easy command of at least one foreign language, and a knowledge of simple book-keeping. Salaries for such work range from £60 to £100 or £120 a-year. For the more highly paid positions good business capacity or special knowledge of some branch of industry is generally requisite. There is also a good demand for working matrons who are willing to do some household work, such as cooking, besides superintendence, and the demand is likely to increase, the present tendency being both in voluntary and poor-law institutions to have small homes rather than large ones. It is added that "there is an extremely small demand for women as matrons who desire superintendence only." To this might be added that even for such a special training is requisite. Merely to have sat at the head of one's father's table is not enough.

Laundry workers and lady servants are also in demand, the demand being greater than the supply. Yet the salary for an assistant manager in a laundry is about thirty shillings, that of a head manager about two pounds weekly, sometimes with a commission on the profits. Lady cooks get from £25 to £40 annually, house parlourmaids £20 to £25, and lady nurses £22 to £25. The report speaks of the demand, small as yet but likely to increase, for women as house decorators. In this originality and talent may gain high pay, but the bulk of workers, even though they may possess skill and artistic feeling, will probably always be lacking in originality. Therefore we should have been glad of information as to the prospects of such women in the decorative work of potteries, in photography, as designers for calico-printers and makers of wall-papers, as designers of dress and illustrators of it, and also as designers of advertisements. Notice is taken of the opening for educated women as teachers in elementary schools. The salaries for such work are good, and the work, once obtained, is very secure, but it must be remembered that training is necessary. A very good governess may make a bad teacher in a school through not being used to manage large classes. Teachers for specially afflicted children, such as the feeble-minded and deaf mutes, are well paid, but must be specially trained for the work. There are still openings, though not so numerous as they were, for cookery teachers, while the demand for teachers of laundry work is likely to increase. Teachers of dairy work are also in request in certain localities.

The authorities of the bureau seem to think that the demand for teachers of hand-loom weaving is likely to increase. We do not agree with them. Hand-loom weaving may be revived as a fad, but never again as a flourishing industry, and girls whose ancestors have been hand-loom weavers for many generations are now anxious to get employment in less decaying trades. Among occupations not mentioned, but which have a future before them at varying rates of pay, may be mentioned commercial travellers (many good bookselling firms employ women canvassers, and there are various drapery specialities where women agents are preferred), druggists and dispensers for medical men, and printers. Good housekeepers, with some of the tact of the successful hostess, earn large salaries as managers of hydropathics. Only one railway company so far as we know (the Caledonian) yet employs women as booking clerks, but it is probable that before long the number will be increased.

